# From laboratory experiments to geophysical tsunamis generated by subaerial landslides

**DOI:** 10.1038/s41598-021-96369-6

**Published:** 2021-09-16

**Authors:** Manon Robbe-Saule, Cyprien Morize, Yann Bertho, Alban Sauret, Anthony Hildenbrand, Philippe Gondret

**Affiliations:** 1 Université Paris-Saclay, CNRS, Laboratoire FAST, 91405 Orsay, France; 2 Université Paris-Saclay, CNRS, Laboratoire GEOPS, 91405 Orsay, France; 3 Department of Mechanical Engineering, University of California, Santa Barbara, CA 93106 USA

**Keywords:** Fluid dynamics, Natural hazards

## Abstract

Modeling of tsunami waves generated by subaerial landslides is important to provide accurate hazard and risk assessments in coastal areas. We perform small-scale laboratory experiments where a tsunami-like wave is generated by the gravity-driven collapse of a subaerial granular column into water. We show that the maximal amplitude reached near-shore by the generated wave in our experiments is linked to the instantaneous immersed volume of grains and to the ultimate immersed deposit. Despite the differences in scale and geometry between our small-scale experiments and the larger-scale geophysical events, a rather good agreement is found between the experimental law and the field data. This approach offers an easy way to estimate the amplitude of paleo-tsunamis.

## Introduction

The tsunami generation by landslides is one of the grand challenges in environmental fluid mechanics^1^. Such events may occur in oceans^2^ but also in lakes^3,4^ or rivers^5^, with different mobilized materials such as soil, rocks, ice, snow^6^ or ash in pyroclastic flows^7^. Small landslides and cliff collapses are frequent but volcanic islands constitute highly unstable reliefs, which also experience recurrent phases of lateral destabilization and may generate highly destructive tsunamis when entering into the ocean. For instance, the Anak Krakatau in Indonesia partially collapsed in 2018, as predicted shortly before by Giachetti et al.^8^. The $0.3~\hbox {km}^3$ landslide generated a tsunami wave of several tens of meters that led to human victims along the neighboring coasts^2^. Many other volcanic islands are prone to such a collapse with an associated risk of tsunamis such as La Réunion in the Indian Ocean^9^ or La Palma in the Atlantic Ocean^10,11^. From inland and offshore studies, past giant landslides with individual volumes reaching a few cubic kilometers to hundreds of cubic kilometers have been identified worldwide^12–16^ that would have corresponded to mega-tsunamis.

The simplest approach to model experimentally the wave generated by a subaerial landslide is the vertical fall of a solid block following the pioneering work of Russell^17^. In this configuration, the amplitude of the leading wave increases non-linearly with the falling mass. Another simplified approach relies on the horizontal displacement of a piston^18,19^. It was found that the amplitude of the generated wave relative to the water depth, $A/h_0$, increases about linearly with the Froude number ${\mathrm{Fr}}=v_0/\sqrt{g h_0}$, corresponding to the ratio of the velocity of the piston $v_0$ to the velocity $\sqrt{gh_0}$ of gravity waves in shallow water of depth $h_0$. However, these crude models do not take into account the granular nature of the landslide and its deformation when entering into water. Experiments have thus been performed with granular material impacting the water surface at high velocity from a pneumatically launched box along a smooth inclined plane^20–25^. This configuration leads to complex scaling laws for the wave generated. The wave amplitude scales mainly with the Froude number based on the grain velocity, but also depends on other parameters such as the thickness of the slide, the slope angle, and the launched mass. All these parameters are sometimes gathered in a so-called “impulse product parameter”^24^. An inclined plane configuration was also considered with a granular mass falling by gravity into water^26–29^. By releasing the granular mass just above the water surface, Viroulet et al.^27^ have found that the wave amplitude increases almost linearly with the falling mass and also increases with the slope angle as the velocity of the slide increases with the slope. When the granular mass is released much further from the water surface^28^, the granular slide entering into water is thin and the wave amplitude is found to scale roughly with the impulse product parameter. Recently, Robbe-Saule et al.^30,31^, Si et al.^32^, Huang et al.^33^, and Cabrera et al.^34^ conducted experiments and numerical simulations on the generation of waves by the gravity-driven collapse of a granular column into a water layer of constant depth. The collapse dynamics of a granular column onto a horizontal bottom has been the subject of an intense research activity in the last years^35–48^ either experimentally^35–37,41,44,45,47^ or numerically^38–43^, and in either dry^35–44^ or immersed cases^44–48^, with evident relevance in geophysical situations^49^. These works show the important role played by the aspect ratio of the granular column on the scaling laws for the deposit, especially for the run-out length. Concerning the coupling of the granular collapse with the surface deformation of a water free surface, Cabrera et al.^34^ demonstrated that the wave amplitude is governed by the submerged fraction of the initial granular column in a small setup. In a large experimental setup, Huang et al.^33^ observed three different kinds of waves similar to those reported by Fritz et al.^20^, depending on the aspect ratio of the column and its submerged fraction. In a medium-scale setup, Robbe-Saule et al.^31^ have shown that the wave amplitude rescaled by the water depth scales almost linearly with a local Froude number based on the horizontal velocity $v_f$ of the granular front at the water surface. Except in the particular case where the granular material is very close in density with water such as in snow avalanches, Robbe-Saule et al.^31^ also highlighted that the density of the particles has a negligible influence on the wave amplitude, suggesting that the volume of the landslide is a key parameter for the generated tsunami^50–52^.

**Figure 1:**
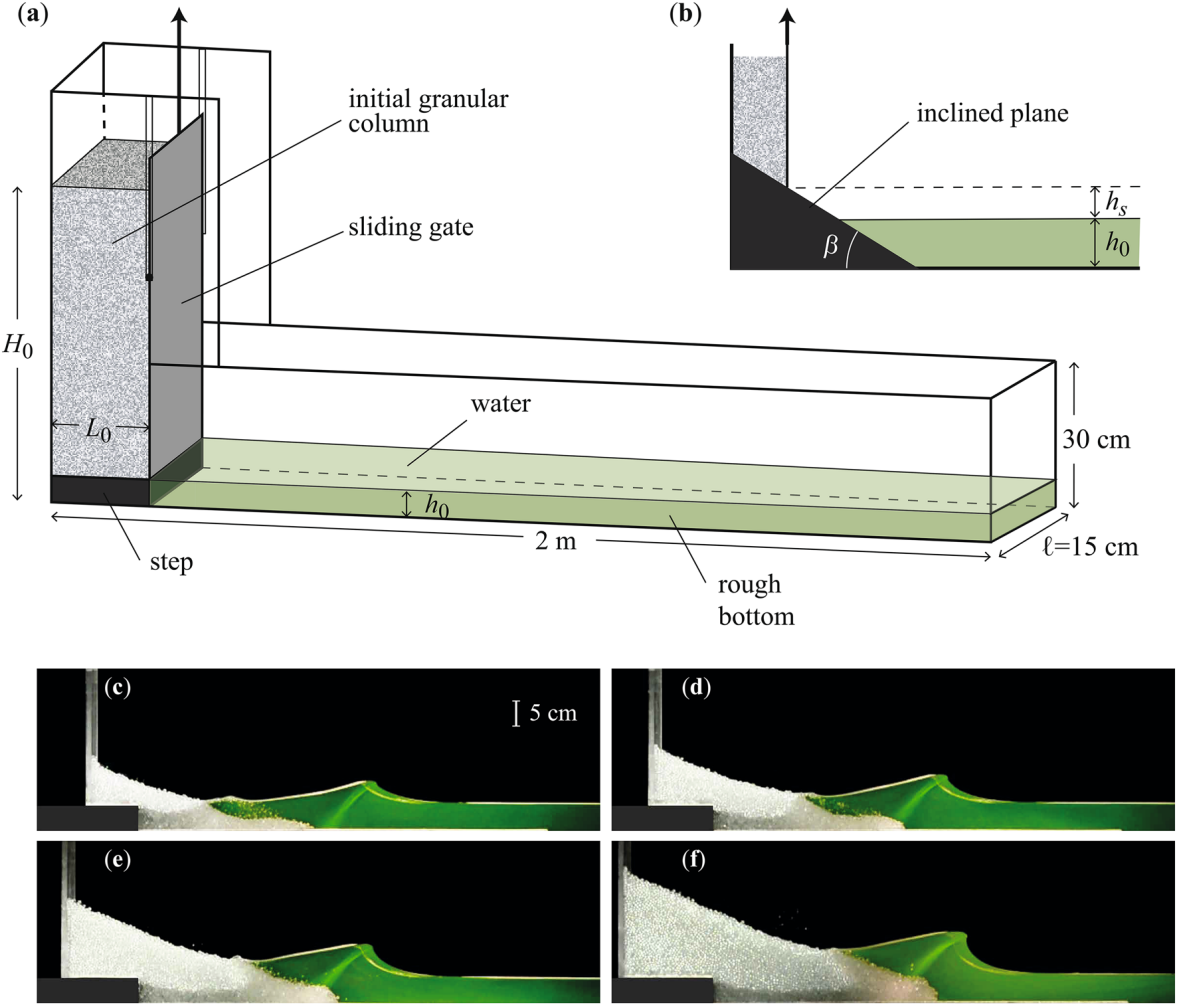
Sketch of the experimental setup for the gravity-driven collapse of initially dry grains over (**a**) a horizontal bottom (setup I) or (**b**) an inclined plane (setup II) into a water layer of depth $h_0$ within a rectangular tank of width $\ell$. (**c–f**) Pictures showing the maximal wave generated by the collapse of an initial column of aspect ratio $a = 2.5$ with different volume $V_g$ of glass beads ($d = 5~\hbox {mm}$, $\rho _g = 2.5~\hbox {g}/\hbox {cm}^3$) in a $h_0 = 5~\hbox {cm}$ water layer for (**c**) $V_g=2.0\,\hbox {dm}^3$ ($\mathrm {Fr}_0 \simeq 2.0$), (**d**) $V_g=3.3\,\hbox {dm}^3$ ($\mathrm {Fr}_0 \simeq 2.3$), (**e**) $V_g=4.9\,\hbox {dm}^3$ ($\mathrm {Fr}_0 \simeq 2.5$), and (**f**) $V_g=7.8$ $\hbox {dm}^3$ ($\mathrm {Fr}_0 \simeq 2.8$).

Here, we focus on the scaling of the wave amplitude with the final immersed deposit from our results of small-scale experiments and of large-scale geophysical events reported in the literature. In the next section, we present our results obtained from the collapse of a subaerial granular column into water using the same experimental setup as Robbe-Saule et al.^30,31^ and a slightly different configuration where the granular column collapses down an inclined plane. A scaling law is obtained between the maximal amplitude of the generated leading wave relative to the water depth and the dimensionless final immersed deposit. Finally, we compare the experimental scaling with data extracted from the literature for several past geophysical events. Despite the large range of volumes and different local topographies, a rather good agreement is observed.

## Experiments

### Experimental methods

Our approach does not aim at reproducing all the details of a real topography that would be specific to a particular geophysical event. Instead, we focus on the key ingredients to improve our understanding of the main parameters involved in the complex process of wave generation: the gravity-driven fall of an initially subaerial granular material into water in a quasi 2D configuration. We focus on the wave generation to characterize the relevant parameters for the maximal “near-shore” amplitude, and not on the far-field wave propagation or inundation and run-up processes. Our setup, shown in Fig. 1a, consists of a glass tank (2 m long, 30 cm high, and $\ell = 15$ cm wide) with a water layer of constant depth $h_0$. At the left-hand side of the tank, a column of height $H_0$ and of length $L_0$ is prepared with dry glass beads on a solid step of height $h_0$ so that the bottom of the granular column coincides with the water surface. The vertical gate, which initially retains the grains, is quickly lifted at $t=0$ without perturbing the water surface. The granular collapse into water together with the wave generation are recorded by a video camera (resolution of $1920 \times 1080$ pixels and acquisition frequency of 25 Hz) placed about 2.5 m from the side wall. The corresponding typical instantaneous images are shown in Fig. 1c-f. More than 40 experiments have been performed varying the height and the length of the column in the range $20 \leqslant H_0 \leqslant 50$ cm and $5 \leqslant L_0 \leqslant 20~\hbox {cm}$, respectively, corresponding to an aspect ratio $a=H_0/L_0$ in the range $1.4 \leqslant a \leqslant 9$ and a volume of grains $V_g$ = $(H_0-h_0) L_0\ell$ in the range $2.0 \leqslant V_g \leqslant 8.8$ $\hbox {dm}^3$. We have also varied the size *d* and density $\rho _g$ of the grains in the range $1 \leqslant d \leqslant 8~\hbox {mm}$ and $1.03 \leqslant \rho _g \leqslant 7.8~\hbox {g}/\hbox {cm}^3$, respectively, and the water depth in the range $2 \leqslant h_0 \leqslant 25~\hbox {cm}$. In this geometry, the global Froude number ${\mathrm{Fr}_0}=\sqrt{H_0/h_0}$, defined as the ratio of the typical vertical free-fall velocity $\sqrt{g H_0}$ of the grains to the typical velocity $\sqrt{gh_0}$ of the gravity waves in shallow water conditions, has been varied in the range $1.8 \leqslant \mathrm {Fr}_0 \leqslant 4.5$. Figure 1c–f show that the amplitude of the generated wave increases with the volume of the released subaerial grains, *V*, and the corresponding global Froude number $\mathrm {Fr}_0$. Note that the wave amplitude is also correlated to the slide thickness which is interrelated to the slide volume. Six additional experiments have also been done with a slightly modified setup sketched in Fig. 1b, where the initial granular column is placed on a smooth plane inclined by an angle $\beta$. In this second geometrical configuration similar to the one of Viroulet et al.^27^, the angle of the inclined plane has been varied in the range $15^{\circ } \leqslant \beta \leqslant 60^{\circ }$ and the height $h_s$ of the bottom edge of the granular column above the water surface has been varied in the range $0 \leqslant h_s \leqslant 8~\hbox {cm}$, with a corresponding sliding length $\ell _s = h_s / \sin \beta$ in the range $0 \leqslant \ell _s \leqslant 15.4~\hbox {cm}$. In this second experimental configuration, the water depth is not constant near-shore, which is closer to geophysical cases. Typical movies of experiments are available in Supplementary Information. For both setups, no significant motion occurs in the transverse direction perpendicular to the lateral walls so that the experiment can be considered as quasi-two-dimensional: we have checked that the width of the tank was large enough to avoid lateral wall effects^53^. By image processing, the instantaneous contour of the granular collapse and the water surface at the sidewall are extracted, from which we determine the instantaneous amplitude *A*(*t*) of the crest of the leading wave above $h_0$ and the length $\lambda (t)$ taken at mid-amplitude *A*/2 as already defined in Robbe-Saule et al.^31^, as well as the instantaneous effective volume $V_{im}(t)$ of grains fallen below the initial unperturbed water surface.

## Results

**Figure 2:**
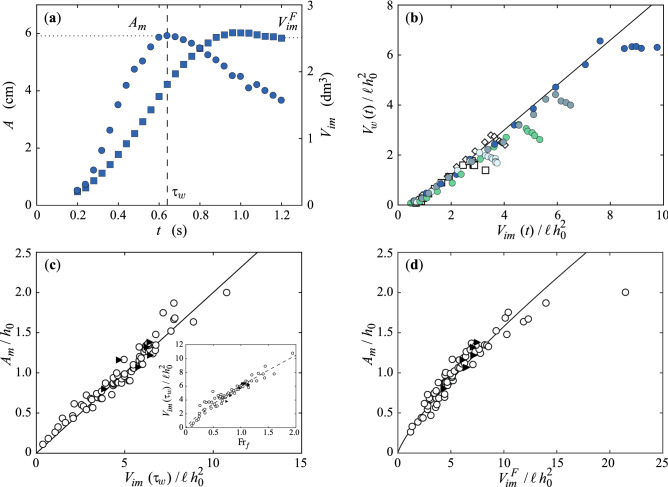
(**a**) Time evolution of () the amplitude *A* of the impulse wave and of () the volume of grains $V_{im}$ entering into water for the experiment shown in Fig. 1e. The vertical dashed line corresponds to the time $\tau _w = 0.65~$ s at which *A* reaches its maximal value $A_m \simeq 6~\hbox {cm}$ in this experiment. (**b**) Instantaneous volume of the impulse wave, $V_w(t) = A(t)\lambda (t)\ell$, rescaled by $\ell h_0^2$ as a function of the instantaneous volume of immersed grains, $V_{im}(t)$, also rescaled by $\ell h_0^2$, for the experiments shown in Fig. 1c–f with a water depth $h_0\, = 5~\hbox {cm}$ and a volume of grains $V_g = 2.0\,\hbox {dm}^3$ (), $3.3~\hbox {dm}^3$ (), $4.9~\hbox {dm}^3$ (), and $7.8~\hbox {dm}^3$ (), and two other experiments with $V_g = 6.2\,\hbox {dm}^3$ and $h_0 = 8 \,\hbox {cm}$ () and 10 cm (). The solid line corresponds to a linear fit of equation $V_w(t)/\ell h_0^2 = 0.9\,V_{im}(t)/\ell h_0^2-0.6$. (**c**) Rescaled maximal amplitude of the impulse wave, $A_m/h_0$, as a function of the corresponding rescaled instantaneous volume of immersed grains, $V_{im}(\tau _w)/\ell h_0^2$, at the time $t=\tau _w$ for all the experiments made either with setup I () or with setup II (). The solid line is a linear fit of equation $A_m/h_0 = 0.2\, V_{im}(\tau _w)/\ell h_0^2$. Inset: $V_{im}(\tau _w)/\ell h_0^2$ as a function of the local Froude number ${\mathrm {Fr}}_f = v_f/\sqrt{g h_0}$ with the same data symbols and (- - -) best fitting power law of equation $V_{im}(t)/\ell h_0^2 = 6\, {\mathrm {Fr}}_f^{0.8}$. (**d**) $A_m/h_0$ as a function of the rescaled final volume of immersed grains, $V_{im}^F/\ell h_0^2$, for all the experiments with the best power law fit of equation $A_m/h_0 = 0.25 (V_{im}^F/\ell h_0^2)^{0.8}$ (—) with a correlation coefficient $R^2 \simeq 0.95$.

Figure 2a reports a typical time evolution of the amplitude *A* of the generated wave and of the instantaneous volume $V_{im}$ of immersed grains for the experiment corresponding to Fig. 1e. The amplitude increases during the wave generation process until it reaches a maximum value $A_m$ at the time $\tau _w$ ($A_m \simeq 6$ cm, and $\tau _w \simeq 0.65$ s for the example in Fig. 2a). Beyond $\tau _w$, the upstream face of the wave is too steep in the example of Fig. 2a so that the wave breaks and, as a result, its amplitude decreases. The wave breaking occurs when the slope of its upstream face, i.e. the inclination of the water surface to the horizontal^31^, exceeds the critical value of 0.34, which is close to the value 0.32 reported by Deike et al.^54^ and corresponds to a critical amplitude-to-depth ratio $A_m/h_0 \simeq 0.7$. The volume of immersed grains $V_{im}(t)$ increases continuously up to a final value $V_{im}^F$ reached when the collapse is over ($V_{im}^F \simeq 2.6$ $\hbox {dm}^3$ in the example of Fig. 2a). All the grains do not fall into water since $V_{im}^F < V_g$ ($V_g = 4.9$ $\hbox {dm}^3$ for Fig. 2a). Furthermore, as the formation of the leading wave is over at $\tau _w$ while the collapse is not yet completed, all the grains which have finally fallen into water do not contribute to the generation process of the leading wave: $V_{im}(\tau _w) \simeq 1.8\ {\mathrm {dm}}^3 < V^F_{im}$ in Fig. 2a.

The instantaneous volume of the impulse wave, $V_w\simeq A\lambda \ell$, normalized by $\ell h_0^2$ is plotted in Fig. 2b as a function of the instantaneous immersed volume of grains $V_{im}$ also normalized by $\ell h_0^2$, for the four experiments of Fig. 1c–f corresponding to different volumes of grains $V_g$ and for two other experiments with different water depth $h_0$. For all the experiments, $V_w(t)$ is proportional to $V_{im}(t)$ during the wave generation process for $t \leqslant \tau _w$. The measurements are captured by the empirical law $V_w(t)/\ell h_0^2\simeq 0.9\,V_{im}(t)/\ell h_0^2 -0.6$, very close to a simple linear scaling $V_w(t) = V_{im}(t)$. For $t > \tau _w$, the data deviate from such a scaling. Indeed, the generation of the leading wave is completed and the grains still falling into water do not contribute anymore to the generated leading wave. The master curve observed for $t \leqslant \tau _w$ suggests that the volume of grains falling into water, resulting from the dynamics of the collapse occurring over the time scale $\tau _w$, drives the dynamics of the wave generation and the maximum wave amplitude.

Figure 2c reports the maximal amplitude of the generated wave rescaled by the water depth, $A_m/h_0$, as a function of the immersed volume of grains $V_{im}(\tau _w)$ at $t = \tau _w$ rescaled by $\ell h_0^2$. We observe a good collapse of all the experimental results obtained with both setup on a master curve of equation $A_m/h_0 \simeq 0.2\, V_{im}(\tau _w)/\ell h_0^2$. Robbe-Saule et al.^31^ have demonstrated that the rescaled amplitude of the wave is also governed by a local Froude number, ${\mathrm{Fr}}_f=v_f/\sqrt{g h_0}$ based on the ratio of the horizontal velocity $v_f$ of the moving granular front at the interface to the velocity of gravity waves in shallow water, which is $\sqrt{gh_0}$ when ignoring the small effect of the wave amplitude on the wave velocity. The inset in Fig. 2c shows that $V_{im}(\tau _w)/\ell h_0^2$ increases with the local Froude number ${\mathrm{Fr}}_f$. The fact that the same scaling law is observed for both configurations suggests that the relations are not specific of a given geometry.

These results obtained from laboratory experiments cannot be tested on geophysical events of much larger scale. Indeed, no data for the instantaneous immersed volume of the landslide during the wave generation is available. However, field data for final immersed deposits can be found in the literature. By now investigating a possible correlation of the wave amplitude with the value of the final immersed volume of grains $V_{im}^F$, the experimental results reported in Fig. 2d show a good collapse onto a master curve that deviates slightly from a linear scaling. The best power fitting law is

1 $$\begin{aligned} \frac{A_m}{h_0} \simeq 0.25 \left( \frac{V_{im}^F}{\ell h_0^2} \right) ^{0.8}, \end{aligned}$$

with a scattering of the data of about 25%. In this weakly non-linear relation, the predicted amplitude of the wave, $A_m$, is zero when the immersed deposit $V_{im}^F$ vanishes, as expected. If a given immersed deposit $V_{im}^F$ is considered (per unit width $\ell$), the amplitude of the wave $A_m$ predicted by Eq. (1) scales as $h_0^{-0.6}$, implying smaller amplitude $A_m$ for larger water depth. Note that $A_m$ would not diverge at vanishing water depth, but would vanish as $V_{im}^F$ would vanish with $h_0$. For very large relative immersed volumes corresponding to very large Froude numbers, there is some deviation from this law as the leading wave starts propagating much before the end of the granular collapse. In the next section, we compare this scaling law with data extracted from the literature dealing with geophysical events occurring at much larger scale.

## Comparison with geophysical events

Numerous past geophysical events are reported in the literature, but only a few contain enough information to allow a comparison with the scaling law (1). Indeed, using this expression requires knowing the water depth $h_0$, the width $\ell$ of the subaerial landslides and the volume of the final immersed deposit $V_{im}^F$. These information may exist from bathymetric measurements and Digital Elevation Model (DEM) pre- and post-event. For the water depth $h_0$, we have taken the value corresponding to the run-out of the immersed deposit. For the width $\ell$ of the slide, we have considered the total width reported at the water surface or sometimes a smaller width corresponding to the main debris mass. But one must know also the near-field maximal amplitude of the generated wave, which is the most challenging point as no direct measurement of $A_m$ exists in the field. We extracted the indirect estimates of the near-field wave amplitude obtained through either far-field measurements, numerical simulation or reduced-scale experiments reported in the literature. These different approaches may lead to significantly different estimates. The experimental geometry used in our study suggests focusing on tsunami waves induced by subaerial landslides. The different field cases reported in Table 1 display a broad data collection in the range $2 \times 10^5\ {\mathrm {m}}^3 \lesssim V_{im}^F \lesssim 3\times 10^8\ \mathrm {m}^3$, $10^2\ \mathrm {m} \lesssim \ell \lesssim 2 \times 10^3\ \mathrm {m}$, $40\ {\mathrm {m}}\lesssim h_0 \lesssim 800\ \mathrm {m}$, and $5\ {\mathrm {m}} \lesssim A_m \lesssim 150\ \mathrm {m}$

**Table 1:** Summary of field data extracted from the literature for 9 subaerial landslides and the recent partially submerged collapse of the Anak Krakatau volcano (2018) that have generated a tsunami wave. For each event and reference paper are indicated the mobilized volume of the landslide *V*, volume of the final immersed deposit $V_{im}^F$, typical lateral extent $\ell$, typical water depth $h_0$, and estimated maximal amplitude $A_m$ of the wave generated near-shore. The last column corresponds to the maximal amplitude $A_{m}^{(1)}$ calculated with the scaling law (1). Data symbols correspond either to () observations in the field, (, ) experiments at reduced scale, (, ) numerical modeling of subaerial landslides, and ($\varvec{*}$) numerical simulations of initially partially submerged landslide.

Events	References	*V*	$V_{im}^F$	$\ell$	$h_0$	$A_m$	$A_{m}^{(1)}$
	(data symbol)	($\times 10^6$ $\hbox {m}^3$)	($\times 10^6$ $\hbox {m}^3$)	(m)	(m)	(m)	(m)
**Subaerial landslides**							
**Askja Lake** (Iceland, 2014)	() Gylfadóttir et al.^56^	20	10	550	120	$>40$	36
	()Ruffini et al.^57^	20	10	550	120	34.7	36
**Chehalis Lake** (Canada, 2007)	() Bregoli et al.^62^	3	3	210	175	18	24
	() McFall and Fritz^63^	3	3	$485 \rightarrow 210^*$	175	$23 \rightarrow 16^\dag$	$7 \rightarrow 24$
**Gongjiafang** (China, 2008)	() Xiao et al.^61^	0.38	0.38	160	120	6	7
**Hongyanzi** (China, 2015)	() Xiao et al.^58^	0.23	0.23	110	40	11	12
**Karrat Fjord** (Greenland, 2017)	() Chao et al.^65^	75	75	1100	430	80	48
	() Paris et al.^66^	53	53	1000	860$^\ddag$	40	26
**Lago Cabrera** (Chile, 1965)	() Watt et al.^59^	$21 \pm 6$	$9 \pm 3$	1000	100	25	23
**Lituya Bay** (Alaska, 1958)	() Fritz et al.^20^	30.6	30.6	338	122	151	129
**Scilla** (Italy, 1783)	() Mazzanti and Bozzano^60^	5.4	5.4	280	300	$<20$	22
	() Zaniboni et al.^67^	5.4	5.4	280	300	$>10$	22
**Taan Fjord** (Alaska, 2015)	() Higman et al.^55^	76	51	830	100	100	107
**Partially submerged landslides**							
**Anak Krakatau** (Indonesia, 2018)	() Grilli et al.^2^	270	$270 \rightarrow 100^\S$	1900	250	50	$121 \rightarrow 54$

$^*$The total width was 485 m but the width corresponding to the main debris mass was 210 m as reported by Roberts et al.^64^.

$^\dag$These values of $A_m$ have been calculated with equation (15) of McFall and Fritz^63^ using the *x* position given by Bregoli et al.^62^ for the maximum amplitude.

$^\ddag$Paris et al.^66^ increased the water depth by a factor of two, calling into question the bathymetric data of Jakobsson et al.^69^.

$^\S$ A volume of $170 \times 10^6~\hbox {m}^3$ was initially immersed so that only $100 \times 10^6\,\hbox {m}^3$ of subaerial material fall into water.

.

**Figure 3:**
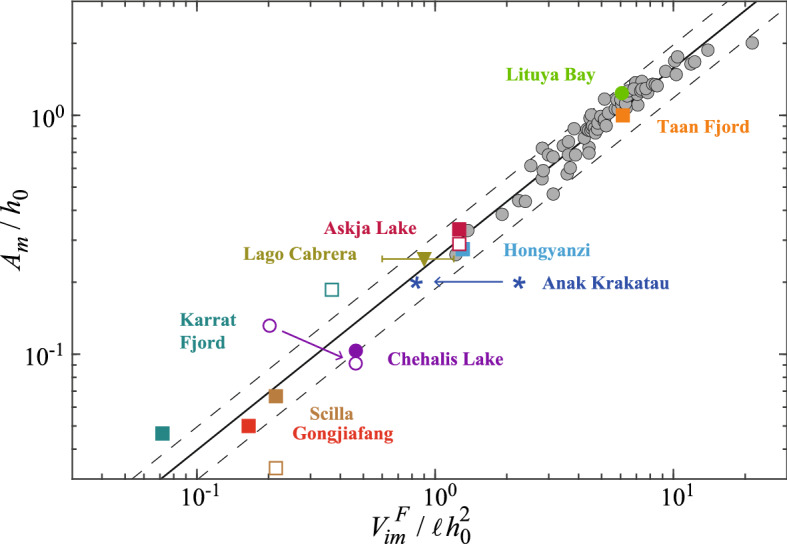
Rescaled maximal amplitude of the wave generated near-shore, $A_m/h_0$, as a function of the rescaled volume of the final immersed deposit $V_{im}^F/\ell h_0^2$ for our experimental data (gray symbols) and data from the literature corresponding to the geophysical events reported in Table 1 (color symbols). The solid line corresponds to the scaling law (1) and the dashed lines indicate a deviation of $\pm 25 \%$ from that law.

The rescaled wave amplitude $A_m/h_0$ of all the geophysical events of Table 1 are plotted in Fig. 3 as a function of the rescaled immersed deposit $V_{im}^F/\ell h_0^2$. Half of these events exhibit smaller $V_{im}^F/\ell h_0^2$ (and $A_m/h_0$) when compared to our laboratory experiments which are in the range $1 \lesssim V_{im}^F/\ell h_0^2 \lesssim 20$ and $0.3 \lesssim A_m/h_0 \lesssim 2$. The largest rescaled field events correspond to the case of Lituya Bay and Taan Fjord with $V_{im}^F/\ell h_0^2 \simeq 6$. For these two events, corresponding to the middle of our experimental range, the agreement for $A_m/h_0$ obtained by a numerical modeling by Higman et al.^55^ for Taan Fjord () and by a reduced scale experiment by Fritz et al.^20^ for Lituya Bay () is very good when compared to our scaling law (1). The agreement is also good for the geophysical events corresponding to the bottom of our experimental range ($V_{im}^F/\ell h_0^2 \simeq 1$), as shown by the three data points from the numerical modeling of Gylfadóttir et al.^56^ and Ruffini et al.^57^ for the Askja Lake event (, ), and of Xiao et al.^58^ for the Hongyanzi event (), and by the data point from the field observations of Watt et al.^59^ for the Lago Cabrera event (). When extrapolating our empirical law for smaller rescaled final deposits ($V_{im}^F/\ell h_0^2 \lesssim 1$), the agreement is sometimes less good, but there is a large dispersion in the reported values from the literature. The agreement is rather good with the data point from the numerical modeling of Mazzanti and Bozzano^60^ for the Scilla event (), and for the numerical modeling of Xiao et al.^61^ for the Gongjiafang event (), and also for the data points from the reduced scale experiments of Bregoli et al.^62^ and of McFall and Fritz^63^ for the Chehalis Lake event (, ) when considering a reduced slide width $\ell$ corresponding to the main debris mass^64^. The agreement is less good for the two numerical modeling of () Chao et al.^65^ and of () Paris et al.^66^ for the Karrat Fjord event, which consider very different slide volume and water depth. The agreement is also less good with the numerical modeling of Zaniboni et al.^67^ for the Scilla event (). Some of these discrepancies may come from the fact that the landslides are sometimes modeled numerically with a simple solid block approach. Some discrepancies may also come from significant 3D effects, e.g. for Karrat Fjord and also for Chehalis Lake.

Recently, Grilli et al.^2^ performed 3D numerical simulations to reproduce the collapse of the Anak Krakatau in 2018 and the resulting tsunami wave. From an estimate of the landslide volume $V= 0.27~\hbox {km}^3$ based on a combined landslide-source and bathymetric datasets, their numerical simulations with two different landslide rheologies, granular and fluid, lead to $A_m \simeq 50~\hbox {m}$. By considering such a landslide volume corresponding to $V_{im}^F/\ell h_0^2 \simeq 2.3$, our scaling law (1) leads to a much larger value $A_m \simeq 121~\hbox {m}$. However, considering only the subaerial part of the landslide of volume $0.1~\hbox {km}^3$ corresponding to $V_{im}^F/\ell h_0^2 \simeq 0.84$, our scaling law (1) leads to the much closer value $A_m \simeq 54~\hbox {m}$. This suggests that the submarine part of the landslide plays a negligible role in the tsunami generation, compared to the subaerial part as demonstrated experimentally by Cabrera et al.^34^ and numerically by Clous and Abadie^68^.

## Conclusion

In this paper, we have investigated the generation of a tsunami-like wave by the collapse of an initially dry column of grains into water in a small-scale laboratory set-up corresponding to a quasi-two-dimensional configuration. The results show that the maximum amplitude of the leading wave generated near-shore rescaled by the water depth scales linearly with the rescaled immersed volume of grains at that time, and weakly non-linearly with the rescaled volume of the final immersed deposit. This experimental scaling law is then tested for ten documented past geophysical events corresponding to subaerial landslides entering into water. Despite large differences in scale and geometry between the laboratory experiments and the geophysical events, a rather good agreement is found between the numerical and experimental modelings from the literature for these events and our scaling law. The maximum amplitude of the near-field wave is predicted with a deviation smaller than 25% in most cases. Our results suggest that a good knowledge of the volume of final deposits associated with a given landslide, e.g. from offshore marine geophysical surveys, can be used to quantify the initial amplitude of the impulse wave, and thus offers an independent and efficient way of estimating the amplitude of paleo-tsunami at first order. However, large-scale experiments and numerical simulations taking into account the real topography specific to a particular event remain essential to obtain more refined results. For partially submerged landslides, such as the collapse of Anak Krakatau volcano that occurred in 2018, we suggest that the subaerial part of the landslide contributes the most to the generation of the wave. In the case of La Palma in Canary Islands, a massive event may occur with a high associated risk^10,11^. Although the subject is debated, when considering the collapse of a volume $V = 80~\hbox {km}^3$ with a lateral extension $\ell =$ 10 km of materials into the sea of depth $h_0 = 4000~\hbox {m}$, similarly to Abadie et al.^11^, we estimate a maximum wave amplitude of the order of 600 m as $A_m/h_0 \simeq 0.15$ for $V_{im}^F/\ell h_0^2 \simeq 0.5$ when extrapolating our scaling law a little below our experimental data range. However, it should be pointed out that a non-negligible part of this volume would be underwater initially so that the actual wave amplitude should be smaller when considering only the aerial falling volume. Potential 3D effects that may be important in the field for events occurring on conical islands^63,70^ will be investigated in a future work through the collapse of cylindrical columns in a square basin.

## Supplementary Information

Supplementary Video 1.

Supplementary Video 2.

Supplementary Video 3.

Supplementary Video 4.

Supplementary Video 5.

Supplementary Video 6.

## Data Availability

All data used in this work are publicly available online (SEANOE. https://doi.org/10.17882/75297).
